# Lemongrass (*Cymbopogon citratus*) Extracts Ameliorate Polycystic Ovary Syndrome in Rats by Inhibiting Follicular Atresia and Modulating Ghrelin and Leptin

**DOI:** 10.1155/bri/5873339

**Published:** 2026-01-11

**Authors:** Vanis Slauvers Akago, Marie Alfrede Mvondo, Sefirin Djiogue, Stephen Lacmata Tamekou, Nina-Sonia Messongue Mbollo, Josue Jobin Biba, Agatha Yelah Ntumnyuy, Lylie Gisèle Atsafack Mboudem, Perpetue Atsama Mbede, Christophe Mezui, Hélène Carole Edima-Durand, Jules-Roger Kuiate, Dieudonné Njamen

**Affiliations:** ^1^ Department of Animal Biology and Physiology, Faculty of Science, University of Yaoundé 1, Yaoundé, Cameroon, uy1.uninet.cm; ^2^ Department of Biotechnology and Pharmacognosy, Faculty of Science, University of Ebolowa, Ebolowa, Cameroon; ^3^ Department of Biochemistry, Faculty of Science, University of Dschang, Dschang, Cameroon, univ-dschang.org; ^4^ Department of Animal Biology, Faculty of Science, University of Dschang, Dschang, Cameroon, univ-dschang.org

**Keywords:** apoptosis, *C. citratus*, ferroptosis, follicular atresia, oxidative stress, PCOS, rats

## Abstract

Polycystic ovary syndrome (PCOS) accelerates ovarian follicular depletion through atresia. This study investigated the therapeutic potential of aqueous (AE) and hydroethanolic (HEE) extracts of *Cymbopogon citratus* in mitigating PCOS‐induced ovarian damage (specifically ferroptosis, apoptosis, and oxidative stress) in a rat model of PCOS. PCOS was induced by oral administration of letrozole (1 mg/kg/day for 21 days), followed by treatment with AE or HEE (100, 200, or 400 mg/kg/day for 20 days) with metformin (200 mg/kg) as a positive control. The results showed that HEE contains 5 times more alkaloids than AE. In experimental animals, *C. citratus* corrected ovarian damage caused by PCOS, compared to the disease control treated with distilled water. Indeed, *C. citratus* decreased serum testosterone levels [maximum decrease rate of 37.10% (*p* < 0.0001) obtained with HEE100] and increased estradiol levels [maximum increase rate of 56.15% (*p* < 0.0001) obtained with AE200]. An increase in serum ghrelin levels [maximum increase rate of 129.38% (*p* < 0.0001) obtained with AE400] and a decrease in leptin levels [maximum decrease rate of 43.85% (*p* < 0.0001) obtained with AE400] were observed. In the ovaries, *C. citratus* decreased Fe^2+^ [maximum decrease rate of 31.57% (*p* < 0.01) obtained with HEE200], caspase 3 [maximum decrease rate of 24.47% (*p* < 0.05) obtained with HEE400], oxidative stress markers [MDA: up to 23.28% decrease (*p* < 0.05) induced by AE100], and increased antioxidants [catalase: up to 29.99% increase (*p* < 0.05) induced by HEE100 and GSH: up to 42.37% increase (*p* < 0.01) induced by HEE200]. *C. citratus* also decreased cystic and atretic follicles and promoted follicle growth and ovulation. In conclusion, *C. citratus* extracts are capable of protecting the ovaries from the adverse effects of PCOS, primarily ferroptosis, and apoptosis of follicular cells. Of the two extracts, AE seems ideal due to its low alkaloid content.

## 1. Introduction

Polycystic ovary syndrome (PCOS) is a prevalent endocrine and metabolic disorder, affecting 8%–10% reproductive‐aged women worldwide, with nearly half experiencing infertility due to anovulation, hyperandrogenism, and polycystic ovarian morphology [1, 2]. Infertility is defined as the inability of a couple to achieve clinical pregnancy after 12 months or more of regular, unprotected sexual intercourse [3]. This reproductive condition affects various aspects of a couple’s life, as the ability to reproduce is closely linked to self‐esteem and sexuality [4].

Indeed, chronic hyperandrogenism induces excess insulin production by pancreatic β cells, which increases the bioavailability of insulin‐like growth factor (IGF)‐1 [5, 6]. IGF‐1 and insulin potentiate the effect of LH on ovarian follicular theca cells, thereby promoting steroidogenesis and mainly androgen production [7, 8]. IGF‐1 and insulin also amplify the effect of LH on granulosa cells, stimulating the early differentiation of these cells, the cessation of follicular growth, anovulation, and the formation of cysts [9, 10].

Ovarian dysfunction associated with PCOS has also been linked to high levels of programmed death of granulosa cells [11–13], which causes ovarian follicle atresia [14–17]. This cell death may be of the ferroptosis type [autophagic cell death characterized by ferrous iron (Fe^2+^)–dependent lipid peroxidation and an increase in cellular reactive oxygen species (ROS) [18–20] or apoptosis [programmed cell death with high caspase 3 levels as a marker] [21, 22]. Under normal conditions, ROS are eliminated by agents called ROS scavengers which include superoxide dismutase (SOD), catalase, reduced glutathione (GSH), and glutathione peroxidase (GPX) [23]. Another molecule with antioxidant capacity is endogenous sulfur dioxide (SO_2_). The latter is a gaseous signaling molecule generated by the oxidation of sulfur‐containing amino acids, such as cysteine and homocysteine, an enzymatic reaction catalyzed by aspartate aminotransferase 1 (AAT1) in mammals [24–26]. Du et al. [27] were the first to identify the SO_2_/AAT pathway in rat cardiovascular tissues. To date, this pathway has been demonstrated in almost all mammalian organs [25]. Endogenous SO_2_ has been shown to possess various cellular functions, including anti‐inflammatory, antioxidant, DNA‐protective, and antiapoptotic effects [26]. Du et al. [28] also reported an inverse association between SO_2_, oxidative stress, and cellular apoptosis.

Two hormones involved in appetite and weight regulation (ghrelin [GHRL] and leptin [LEP]) have also been implicated in impaired ovarian function in animals with PCOS. Indeed, under normal conditions, physiological concentrations of GHRL inhibit the activity of the hypothalamic–pituitary axis by preventing the release of GnRH from hypothalamic neurons and of FSH and LH from the pituitary gland [29, 30]. In the ovaries, GHRL promotes follicular maturation by inhibiting apoptosis [31, 32]. The effects of GHRL are mediated by the growth hormone secretagogue receptor (GHS‐R), which is present in both GnRH neurons [29] and follicles [31, 32]. LEP increases LH by stimulating GnRH release via kisspeptin receptors in arcuate neurons [33] and stimulates follicular development and steroidogenesis at physiological levels [32]. In PCOS, a decrease in GHRL levels and an increase in LEP levels are observed. These changes contribute to elevated LH levels and, consequently, to hyperandrogenism and ovarian dysfunction, as the increase in LH stimulates the overproduction of androgens by the ovaries [32].

Conventional treatments for PCOS, although effective, are mostly symptomatic and are associated with adverse effects that limit their use. This is the case with clomiphene citrate (an ovulation inducer), which, when taken continuously, increases the risk of multiple pregnancies and hot flushes [34], vision problems [35], inhibits implantation [36], and increases the risk of ovarian cancer [37]. Aromatase inhibitors (such as letrozole [LTZ]) were found to have embryotoxic, fetotoxic, and teratogenic potential [38, 39] and to increase the incidence of fatigue and dizziness [38, 40, 41]. Insulin‐sensitizing drugs such as metformin induce lactic acidosis, nausea, bloating, cramps, and diarrhea [38]. Antiandrogens are hepatotoxic and teratogenic [42, 43]. The use of injectable gonadotropins, in addition to being costly, requires close monitoring, frequent ultrasound assessments, and serum estradiol measurement. It was also associated with an increased risk for multiple pregnancies and ovarian hyperstimulation syndrome [38]. Surgical procedures (e.g., ovarian drilling), which are also costly, have been associated with operative morbidity and risk of postoperative adhesions [38, 44].

It is therefore important to develop new treatments for the clinical management of PCOS, treatments that would be more effective and less toxic. Plants with antioxidant and anti‐inflammatory properties are considered potential alternatives to conventional treatments for PCOS [45]. Experimental studies have demonstrated the efficacy of several medicinal plants on the symptoms of PCOS in laboratory animals. These include *Allium fistulosum* [46], *Milicia excelsa* [47], *Allium ampeloprasum* var. porrum [48], *Agaricus subrufescens* [49], and *Rosa damascena* [50]. Several plant‐derived compounds have also shown satisfactory effects on PCOS. These include quercetin [51], curcumin and berberine [52], naringenin [53], and diosmetin [54]. *Cymbopogon citratus* (DC.) Stapf (Poaceae) is a plant in the Cameroonian pharmacopoeia commonly consumed in the form of tea. It has been the subject of numerous scientific studies highlighting its antioxidant [45, 55] and anti‐inflammatory [45, 56] properties. In diabetic rats, aqueous extract (AE) of *C. citratus* improved hyperglycemia and lipid profile, and reduced weight gain [57–59]. Antiparasitic [45] and anticancer [55] properties of this plant were also reported. The classes of chemical compounds found in this plant include polyphenols and alkaloids, which give it antiradical/antioxidant activity [56, 60, 61]. Since the content of active ingredients in a plant varies, sometimes very significantly depending on the stage of development of the plant, but also depending on the region, climate, and weather conditions, as well as the storage time, extraction method, and extraction solvents [62, 63], it is important to assess the chemical composition of a plant to ensure the presence of the ingredients responsible for the desired pharmacological properties. In the present study, we carried out a phytochemical screening of *C. citratus* extracts for the detection of polyphenols (flavonoids, tannins) and alkaloids, classes of compounds known for their ability to trap free radicals [61, 64–66]. Alkaloids are also known to possess insulin‐sensitizing and antiapoptotic properties [65–67].

Moreover, the above‐mentioned properties of *C. citratus* suggest that this plant could effectively counteract ovarian follicle atresia and encystment by correcting ovarian damage (ferroptosis, apoptosis, oxidative stress) caused by PCOS‐related hyperandrogenism. To test this hypothesis, the effects of AE and hydroethanolic (HEE) extracts of *C. citratus* were evaluated on ovarian dynamics, particularly folliculogenesis and steroidogenesis. The latter was estimated through serum testosterone and estradiol levels. In an attempt to elucidate the mechanism of action of these extracts, serum LEP and GHRL levels were evaluated. In the ovaries, markers of ferroptosis (Fe^2+^), apoptosis (caspase 3), and oxidative stress [malondialdehyde (MDA), sulfur dioxide (SO_2_), catalase, and reduced GSH] were evaluated.

## 2. Materials and Methods

### 2.1. Animals

The experiments were conducted on 54 healthy female Wistar rats aged 10–12 weeks, weighing an average of 152 g, with a regular estrous cycle and raised at the animal facility of the Department of Biochemistry, University of Dschang, Cameroon. The estrous cycle was monitored by daily observation of vaginal smears under a microscope for 25 days before PCOS induction, as we described previously [48]. Animals exhibiting three successive normal estrous cycles were considered to have a regular estrous cycle. A normal estrous cycle lasts on average 5 days and includes the following phases: proestrus, estrus, metestrus, and diestrus [48].

Animals were housed in plastic cages (50 cm in diameter and 20 cm in height) (*n* = 6 rats per cage) lined with wood shavings. They were raised at room temperature, with adequate ventilation and a natural light/dark cycle (12:12 h). The animals had free access to water and soy‐free food to avoid interference from phytoestrogens.

### 2.2. Ethics Statement

This study was conducted after approval of the research proposal by the scientific committee of the Department of Animal Biology and Physiology of the University of Yaoundé 1, on October 24, 2023. The Ethical clearance N° BTC‐JIRB2023‐081 was issued by the Joint Institutional Review Committee for Animal and Human Bioethics of the University of Yaoundé 1. The conditions for experimentation and animal handling complied with the European Union Directives 2010/63/EEC relating to animal testing.

### 2.3. Plant Material

#### 2.3.1. Collection and Authentication

*Cymbopogon citratus* was harvested in Fondonera in the Santchou District (Western Region, Cameroon) in September 2024. A sample of this plant was authenticated at the National Herbarium of Cameroon by comparison with the original sample, Voucher No. 31953/IINC, collected by Guarisma. The fresh plant was dried in the shade, in a well‐ventilated place (22°C–25°C, 0.1–0.3 m/s, 5 days) and then ground in a grinder [multifunction blender robots, Silver Crest (SC‐1589)]. The powder obtained was used to prepare AE and HEE of the plant.

#### 2.3.2. Preparation of Extracts

##### 2.3.2.1. Preparation of the AE of *C. citratus*

The AE of *C. citratus* was prepared according to the protocol described by Namekong et al. [56] with slight modifications. Briefly, 150 g of *C. citratus* powder was boiled in 1.5 L of distilled water (DW) for 10 min. After cooling, the mixture was sieved and then filtered through coffee filter paper. The filtrate obtained was dried in an oven at 45°C for 3 days. The crude extract (14.66 g; extraction yield: 9.77%) obtained was stored in a cold place (4°C) until use.

##### 2.3.2.2. Preparation of the HEE of *C. citratus*

To prepare the HEE of *C. citratus*, 150 g of *C. citratus* powder was macerated at room temperature in 1.5 L of water–ethanol solvent (in a ratio of 30:70) for 48 h. At the end of maceration, the mixture was filtered through coffee filter paper. The filtrate obtained was dried in an oven at 45°C for 2 days. The crude extract (23.13 g; extraction yield: 15.42%) obtained was stored in a cold place (4°C) until use.

#### 2.3.3. Justification of the Doses Used and the Duration of Treatment

The doses used (100, 200, and 400 mg/kg) were extrapolated from the work of Nnam et al. [57]. These authors reported that the AE of *C*. *citratus*, administered to diabetic *Wistar* rats at a dose of 400 mg/kg for 21 days, induced antihyperglycemic and hypolipidemic effects. Given that hyperglycemia and dyslipidemia are metabolic disorders observed in PCOS [48, 68, 69], this dose was divided by 2 and 4 to obtain lower doses of 100 and 200 mg/kg.

#### 2.3.4. Determination of the Content of Some Classes of Secondary Metabolites in *C. citratus* Extracts

##### 2.3.4.1. Determination of Total Phenol Content

The total phenol content was determined using the method described by Ramde‐tiendrebeogo et al. [70]. The reagent consisted of Folin–Ciocalteu reagent (a mixture of phosphotungstic acid with the chemical formula H_3_PW_12_O_40_ and phosphomolybdic acid with the chemical formula H_3_PMO_12_O_40_). This reagent is reduced during the oxidation of phenols to a mixture of blue tungsten and molybdenum oxides. These blue pigments have a maximum absorption that varies depending on the qualitative and/or quantitative composition of the phenolic mixtures, in addition to the pH of the solutions, generally obtained by adding sodium carbonate [71]. For this assay, 20 μL of each extract, prepared at a concentration of 2 mg/mL, was introduced into the wells of the microdilution plates, then 100 μL of Folin–Ciocalteu reagent diluted to 1/16th (v/v) was added, and finally, 80 μL of a 20% sodium carbonate solution was added to trigger the reaction. The mixture was incubated at room temperature for 30 min, and then, the absorbance was measured at 765 nm using a spectrophotometer. The blank consisted of the reagents and DW. The total phenol content was calculated using the calibration curve equation for gallic acid, with concentrations ranging from 10 to 70 μg/mL. The results were expressed in milligrams of gallic acid equivalent per gram of extract.

##### 2.3.4.2. Determination of Total Flavonoid Content

The total flavonoid content was determined by colorimetry. In the presence of aluminum trichloride and potassium acetate, the flavonoids contained in the sample react to form a pinkish solution. This pinkish mixture has a maximum absorption at around 510 nm. The total flavonoid content of the extracts was determined according to the experimental protocol described by Chang et al. [72]. To do this, a total volume of 100 μL of each extract (2 mg/mL) was placed in test tubes. Then, 30 μL of NaNO_2_ solution (5%), 1400 μL of DW, and 200 μL of AlCl_3_ solution (10%) were added. The mixture was incubated at room temperature (25°C) for 5 min. After incubation, 200 μL of NaOH solution (10%) was added and the mixture was again incubated at room temperature for 5 min. Finally, 200 μL of DW was added to the tubes. The mixture was stirred, and the optical density was read at 510 nm. The blank consisted of all the reagents and DW. The total flavonoid content was calculated using the calibration curve equation for quercetin, with concentrations ranging from 0.015 to 2 mg/mL. The results were expressed in milligrams of quercetin equivalent per gram of extract.

##### 2.3.4.3. Determination of Total Tannin Content

The total tannin content was determined by the Folin–Ciocalteu method as described by Govindappa et al. [73]. In brief, the reaction mixture in this test consisted of 100 μL of each extract (2 mg/mL), 500 μL of Folin–Ciocalteu reagent (diluted 10 times in water), 1000 μL of a 35% sodium carbonate solution, and 8.4 mL of DW. The mixture was stirred and incubated at room temperature for 30 min, and then, the absorbance was measured at 700 nm using a spectrophotometer. The extracts were replaced with DW in the blank tubes. A calibration curve was plotted using tannic acid (tannic acid concentrations ranged from 100 to 500 μg/mL). The results were expressed in milligrams of tannic acid equivalent per gram of extract.

##### 2.3.4.4. Determination of Alkaloid Content

The total alkaloid content was quantified using a modified liquid–liquid extraction method [74], based on the differential solubility of alkaloids in acidic and alkaline media [75]. Briefly, 2.5 g of each extract was dissolved in 100 mL of 10% acetic acid in 96% ethanol and macerated for 4 h at room temperature. The mixture was then filtered and concentrated using a rotary evaporator (40°C, reduced pressure). Alkaloids were precipitated by dropwise addition of 24.5% ammonium hydroxide until pH ≥ 9. The precipitate was washed with diluted ammonium hydroxide (1:10 v/v in DW), dried to constant weight at 60°C, and weighed. The mass obtained was considered to be the total alkaloid content.

##### 2.3.4.5. Determination of Carbohydrate Content

In an acidic and hot environment, starch is hydrolyzed to glucose and dehydrated to hydroxymethylfurfural. This compound forms a green‐colored product with anthrone. The carbohydrate content in the extracts was determined using anthrone reagent [76]. To do this, 1 mL of the extract solution was taken and placed in a test tube. Next, 4 mL of anthrone reagent was added and the mixture was incubated in a water bath at 95°C for 15 min. After incubation, the mixture was cooled under running water, and the optical density was read at 630 nm. The blank tubes contained DW instead of extracts. The carbohydrate concentration was calculated using a D‐glucose calibration curve with concentrations ranging from 1.56 to 200 μg/mL.

#### 2.3.5. Determination of the Antiradical Activity of *C. citratus* Extracts

##### 2.3.5.1. Test With 2,2‐Diphenyl‐1‐picrylhydrazyl (DPPH)

The DPPH test was performed as described by Menezes et al. [77]. DPPH was prepared by dissolving the DPPH powder in methanol. Briefly, 20 μL of methanol was introduced into each well of a 96‐well plate. This was followed by the introduction of 20 μL of the methanolic solutions from the *Cymbopogon citratus* extract (2 mg/mL). In the first two wells of each column (4 columns were used for one sample), successive dilutions in series of two were made from the second line to the fifth line. A volume of 180 μL of DPPH methanolic solution (0.08 mg/mL) was introduced into each of the wells of the first three columns, while 180 μL of methanol was introduced into each of the wells of the fourth column. The plates containing 200 μL of final solution per well were incubated for 30 min in the dark, and the absorbances (A) were read by the spectrophotometer (FLUOstar Omega microplate reader) at 570 nm. The obtained absorbances were converted into percentage of antioxidant activity. Ascorbic acid was used as a positive control. For each sample, three replicates were performed and the percentages of antioxidant activity in each sample were calculated as follows: %*I* = [(*A*_DPPH—(*A*_sample—*A*_blank))/*A*_DPPH] × 100.

%*I* = inhibition percentage; *A* = absorbance; sample = Methanolic extract + methanolic DPPH solution; Blank = methanolic extract + methanol.

The different percentages of DPPH inhibition were used to determine the concentrations needed to inhibit 50% of the radical DPPH (IC_50_) [78]. To do this, regression lines were plotted using the different percentages of inhibitory activity of DPPH and sample concentrations [%] = *f* [*C*]. The equations of the regression lines (*y* = ax + *b*) were used. The calculation of IC_50_ was done for each extract assuming *y* = 50; hence, the relation IC_50_ = (50‐*b*)/*a*.

The IC_50_ values obtained after the three replications were used to determine the average IC_50_ value.

##### 2.3.5.2. Determination of the Reducing Power of Iron

In test tubes containing previously 100 μL of extract solutions prepared at a concentration of 2 mg/mL, 500 μL of phosphate buffer (0.2 M; pH 6.6) was added, followed by 500 μL of aqueous solution of potassium hexacyanoferrate [K_3_Fe (CN)_6_] 1%. The mixture was incubated for 30 min at 50°C in a water bath, followed by 500 μL of 10% trichloroacetic acid solution. The mixture was centrifuged at 3000 rpm for 10 min. The supernatant was used for absorbance measurement. Ascorbic acid standard (vitamin C) was prepared under the same conditions to compare the reducing power of different extracts. The blank consisted of all reagents except extracts. The absorbance of the reaction mixture was read at 700 nm against that of the blank, and the tests were done in triplicate. The reducing power of the samples was calculated from equation (*Y* = 0.0154*x* + 0.1148) of the regression line of the iron sulfate (FeSO_4_) calibration curve.

### 2.4. Experimental Protocol

#### 2.4.1. Induction of PCOS

PCOS was induced in 48 rats by oral administration of LTZ at a dose of 1 mg/kg for 21 days, as described by Fofie and Mvondo [48]. Rats used as a normal control (*n* = 6) received DW during PCOS induction. Induction of PCOS was confirmed by body weight gain and estrous cycle blockage at the diestrus phase as described by Fofie and Mvondo [48].

#### 2.4.2. Allocation and Treatment of Animals

After induction of PCOS, the animals were randomly divided into 9 groups of six rats each and treated by oral gavage for 20 days as described in Table 1.

**Table 1:** Allocation and treatment of animal.

	Group codes	Groups	Treatment
Normal animals	N (*n* = 6)	Normal control	Distilled water

PCOS animals	Neg (*n* = 6)	Negative control	Distilled water
	Pos (*n* = 6)	Positive control	Metformin (200 mg/kg)
	AE100 (*n* = 6)	AE 100 mg/kg	Aqueous extract of *C. citratus* at the dose of 100 mg/kg
	AE200 (*n* = 6)	AE 200 mg/kg	Aqueous extract of *C. citratus* at the dose of 200 mg/kg
	AE400 (*n* = 6)	AE 400 mg/kg	Aqueous extract of *C. citratus* at the dose of 400 mg/kg
	HEE100 (*n* = 6)	HEE 100 mg/kg	Hydroethanolic extract of *C. citratus* at the dose of 100 mg/kg
	HEE200 (*n* = 6)	HEE 200 mg/kg	Hydroethanolic extract of *C. citratus* at the dose of 200 mg/kg
	HEE400 (*n* = 6)	HEE 400/kg	Hydroethanolic extract of *C. citratus* at the dose of 400 mg/kg

*Note:* Treatments were administered orally to the animals for 20 consecutive days, and animals were then sacrificed under anesthesia for biochemical and histological analyses.

#### 2.4.3. Animal Sacrifice Procedure and Sample Collections

**Figure 1:**
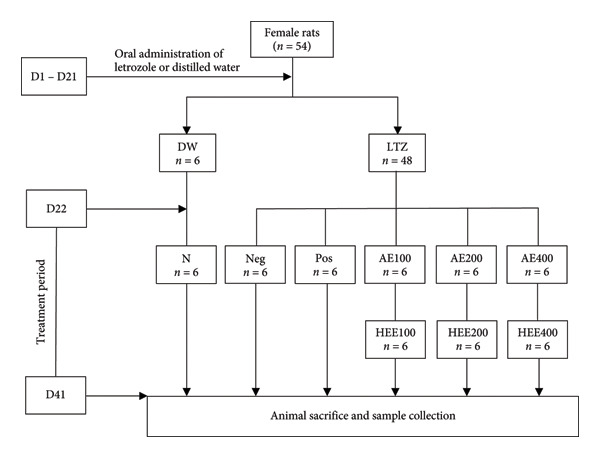
Schematic representation of experimental protocol. AE: aqueous extract; D: day; HEE: hydroethanolic extract; DW: distilled water; LTZ: letrozole; N: normal control; Neg: negative control; Pos: positive control; PCOS: Polycystic ovarian syndrome.

The day following the end of treatment, the animals were anesthetized by intraperitoneal injection of diazepam (10 mg/kg) and ketamine (50 mg/kg). The blood, taken from a catheter in the abdominal artery in dry tubes, was centrifuged at 3000 rpm for 10 min. The serum (supernatant) obtained was kept at −20°C for the determination of biochemical parameters. The ovaries of each animal were collected. The right ovary of each animal was kept at −20°C for the preparation of homogenates and the determination of biochemical parameters. The left ovary was fixed in 10% formalin for histological analysis (Figure 1).

#### 2.4.4. Preparation of Ovarian Homogenates

The ovarian homogenates were made in phosphate buffer (pH 7.4) in the proportions of 10% (w/v). To do this, a portion of the tissue weighing 0.1 g was crushed in 1 mL of phosphate buffer using the Teflon‐glass potter on an ice tray to obtain a final homogenate of 10%. After centrifugation at 3000 rpm/min for 15 min, the collected supernatant was kept at ‐ 20°C for the determination of biochemical parameters.

#### 2.4.5. Hormone Assays

Hormone assays were performed using Elabscience ELISA kits (USA), according to the supplier’s recommendations.

Serum estradiol (E_2_) and testosterone levels were assessed using “QuicKey Pro Rat E_2_ (Estradiol) kit” [sensitivity: 1.17 pg/mL; standard curve range: 3.13–200 pg/mL] and “QuicKey Pro Rat T (Testosterone) kit” [sensitivity: 0.07 ng/mL; standard curve range: 0.16–10 ng/mL], respectively.

Serum GHRL and LEP levels were assessed using “Rat GRHL (Ghrelin) kit” and “Rat LEP (Leptin) kit.” For both kits, the sensitivity was 0.09 ng/mL; the calibration curves ranged from 0.16 to 10 ng/mL.

Absorbance of the standards and samples was measured using a BIOBASE BK‐EL10C ELISA microplate reader (Biobase Meihua Trading Co., Ltd., China). Hormone concentrations were determined from calibration curves established by the calibrators supplied with the kits.

#### 2.4.6. Determination of Markers of Ferroptosis, Apoptosis, and Oxidative Stress in the Ovaries of Experimental Animals

##### 2.4.6.1. Total Protein Assay

The levels of proteins in the ovary homogenates were assessed using the protocol described by Gornall et al. [79]. The absorbance was read against the blank by a spectrophotometer at 540 nm. A linear regression curve model from the EXCEL software was used to obtain the calibration curve. The amount of protein (Cpr) was determined as a function of the sample absorbance by reference to the total protein calibration curve. This parameter was used to evaluate the level of antioxidant enzymes.

##### 2.4.6.2. Fe^2+^ Assay

The level of ferrous iron in the ovary homogenate was assessed using the “ferrous iron (Fe^2+^) assay kit (colorimetric NBP3; 25,791)” [sensitivity: 0.08 mg/L], purchased from NOVUS BIOLOGICAL, a BIOTECHNE Brand (USA). The concentration of ferrous iron in the ovarian homogenates was calculated according to the following formula:

Iron content (mg/g of Prot) = [(OD1/OD2) × C1 × f)]/Cpr; with, OD1: OD sample ‐ OD blank; OD2: OD standard – OD blank; C1: Concentration of the standard (2 mg/L); *f*: sample dilution factor before test; Cpr: protein concentration in the sample (g prot/L); OD: optical density.

##### 2.4.6.3. SO_2_ Ion Assay

The concentration of sulfite ions (SO_2_) in ovarian homogenate was assessed using “total sulfite assay kit” [sensitivity: 20 μmol; standard curve range: 0–10 nmol], purchased from SIGMA ALDRICH (USA). The absorbance of the calibrators and specimens was evaluated using a microplate reader [BIOBASE BK‐EL10C (Biobase Meihua Trading Co., Ltd., China)]. Sulfite SO_2_ concentrations were evaluated using calibration curves established by the calibrators supplied with the kits.

##### 2.4.6.4. Caspase 3 Assay

The concentrations of caspase 3 in ovary homogenates were assessed using the “Rat CASP3 (Caspase 3) ELISA Kit” [sensitivity: 0.19 ng/mL; standard curve range: 0 – 20 ng/mL], purchased from Elabscience (USA). The absorbance of the calibrators and specimens was evaluated using an ELISA microplate reader [BIOBASE BK‐EL10C (Biobase Meihua Trading Co., Ltd., China)]. Caspase 3 concentrations were evaluated using calibration curves established by the calibrators supplied with the kits.

##### 2.4.6.5. MDA Assay

The level of MDA in the ovarian homogenates was assessed according to the protocol described by Wilbur et al. [80]. This assay is based on the principle by which the presence of MDA in a sample result in the formation of aldehydes in hot acid (100°C), including the malonic aldehyde which reacts with thiobarbituric acid (TBA) to form a pink complex that absorbs at 530 nm. This complex accounts for the amount of MDA in the sample. The concentration of MDA was determined using the following formula:

[MDA] (mol/g protein) = (A × Vt)/(*ε* × l × Vi × m), where A: absorbance (nm); Vt: total volume in the tube (mL) = 1.25 mL; Vi: volume of supernatant used for dosage (mL) = 0.5 mL; m: mass of proteins (g) contained in 1 mL of sample; l: length of the vessel = 1 cm; and *ε*: molar extinction coefficient of MDA = 156.10^5^ mol^−1^·cm^−1^.

##### 2.4.6.6. Catalase Assay

The level of catalase in the ovarian homogenates was assessed according to the protocol described by Sinha [81]. The principle of this protocol stipulates that hydrogen peroxide is broken down in the presence of catalase. This destroyed peroxide binds to potassium dichromate to form an unstable blue–green precipitate of perchloric acid, which is then broken down by heat and forms a green complex that absorbs at 570 nm. The catalase activity, which is proportional to the optical density, is determined by a calibration curve. The specific activity of catalase was expressed in mM H_2_O_2_/min/mg in ovarian tissue according to the following formula: catalase activity (mmol of H_2_O_2_/min/g prot) = [(DO_sample_ − DO_Blank_)/*a* × *t* × *m*] × *f*.

Here, DO_sample_ is the optical density of the sample; DO_Blank_ is the optical density of the blank; *F* is the dilution factor = 1/10; *a* is the coefficient of the equation line of the calibration curve = 0.0149; *t* is the reaction time = 1 min; and *m* is the mass of proteins (g) contained in 1 mL of sample.

#### 2.4.7. Histological Analysis

Histology of the ovaries was performed using a microtome (Leica). The ovary sections (5 μm thick) were stained with hematoxylin and eosin, then fixed to slides, and covered with coverslips using Canada balm. Ovarian tissue attached to the slides was filmed using a Leica microscope, equipped with a Celestron MA411101 camera connected to a computer, where the images were transferred and analyzed with Image J1.3 software. The histological sections obtained were used to count (using a Leica microscope) the different types of follicles (tertiary follicles, Graafian follicles, atretic follicles, cystic follicles, and corpora lutea). For an ovary, three sections were considered (the 10^th^, 20^th^, and 30^th^ sections). Thus, for each ovary, the number of a follicular type is the average of the data obtained from the three sections.

### 2.5. Statistical Analysis

Results are expressed as mean ± standard error of the mean (SEM). All analyses were performed using GraphPad Prism Version 8.0.1. During PCOS induction (there were two variables: time and LTZ), body weight data were compared using a two‐way analysis of variance (ANOVA), followed by Tukey’s test (to compare body weight recorded at Week 1 with that of Weeks 2, 3, and 4) and Sidak’s test (to compare each week the average body weight of the animals treated with DW with that of the group treated with LTZ). At the end of the treatment period (there was only one variable: treatments), multiple comparisons (treated groups versus control groups) were performed using a one‐way ANOVA, followed by Tukey’s test. The Brown–Forsythe test and Bartlett’s test were used to verify the hypothesis of equality of variances before proceeding with the one‐way ANOVA. *p*‐values less than 0.05 were considered statistically significant.

### 2.6. Processing of References

All references were generated with Mendeley software (2.79.0).

## 3. Results

### 3.1. Chemical Analysis of *C. citratus* Extracts

The total phenol contents of the AE and HEE of *C. citratus* are shown in Table 2. It comes out from this table that the total phenol content was higher in the HEE (12.40 ± 0.13 mg GAE/g of extract) compared to AE (10.75 ± 1.01). The level of flavonoids was slightly higher in the HEE (7.38 ± 0.12 mg EQ/g extract) than in the AE (6.05 ± 0.24 mg QE/g extract). Similarly, the total tannin content was higher in the HEE (5.82 ± 1.877 mg TAE/g extract) compared to the AE (3.05 ± 0.35 mg TAE/g extract). For alkaloids, the results show that the HEE had an alkaloid content 5 times higher (724 mg) than the AE (144 mg). Carbohydrate levels were almost identical in both extracts.

**Table 2:** Content of phenols, flavonoids, tannins, alkaloids, and carbohydrates in aqueous and hydroethanolic extracts of *C. citratus*.

	TPC (mg GAE/g extract)	TFC (mg QE/g extract)	TTC (mg TAE/100 g dry matter)	TAC (mg)	TCC (mg GE/g extract
AE	10.75 ± 1.01	6.05 ± 0.24	3.05 ± 0.35	144	104.63 ± 12.72
HEE	12.40 ± 0.13	7.38 ± 0.12	5.82 ± 1.877	724	103.12 ± 13.25

*Note:* HEE, hydroethanolic extract.

Abbreviations: AE, aqueous extract; GAE, gallic acid equivalent; GE, glucose equivalent; QE, quercetin equivalent; TAC, total alkaloid content; TAE, tannic acid equivalent; TCC, total carbohydrate content; TFC, total flavonoid content; TPC, total phenol content; TTC, total tannin content.

### 3.2. Antiradical Activity of AE and HEE of *C. citratus*

Table 3 shows that the concentration of the AE (174.31 ± 7.92 μg/mL) inhibiting 50% of DPPH was similar to that of the HEE (176.12 ± 2.45 μg/mL). However, the iron‐reducing power of the HEE (68.30 ± 8.19 μmol of FeSO4/g of extract) was higher than that of the AE (56.70 ± 2.33 μmol of FeSO_4_/g of extract).

**Table 3:** Antiradical activity of aqueous and hydroethanolic extracts of *C. citratus*

	DPPH IC_50_ (μg/mL)	FRAP (μmol of FeSO_4_/g of extract)
AE	174.31 ± 7.92	56.70 ± 2.33
HEE	176.12 ± 2.45	68.30 ± 8.19
Vit C	6.84 ± 1.02	135.56 ± 7.16

*Note:* HEE, hydroethanolic extract; DPPH, 2,2‐diphenylpicryl‐1‐hydrazyl; %I, inhibition percentage; Vit C, vitamin C.

Abbreviations: AE, aqueous extract; FRAP, ferric reducing antioxidant power.

### 3.3. Effects on Body Weight in Animals With PCOS

Figure 2(a) illustrates the evolution of the animals’ body weight during the induction of PCOS. This figure shows that the body weight of the animals receiving DW and that of the animals receiving LTZ were comparable at the start of the experiment: 152.4 ± 1.90 g for the DW group and 152.73 ± 1.03 g for the LTZ group. During the 21 days (3 weeks) of LTZ administration, body weight increased progressively in both groups compared to Week 1. In the DW group, it increased from 152.40 ± 1.90 g in Week 1–154.25 ± 1.07 g in Week 2 (1.21% increase; *p* = 0.2116), 156.25 ± 1.42 g in Week 3 (2.52% increase; *p* = 0.0033), and 160.50 ± 1.22 g in Week 4 (5.31% increase; *p* < 0.0001).

Figure 2Changes in animal body weight during PCOS induction (a) and treatment with *C. citratus* (b). AE: aqueous extract; DW: distilled water; HEE: hydroethanolic extract; LTZ: letrozole; N: normal control; Neg: negative control; Pos: positive control. Results are presented as mean ± SEM. *n* = 6; ^∗∗^ *p* < 0.01, ^∗∗∗^ *p* < 0.001, and ^∗∗∗∗^ *p* < 0.0001 vs Week 1; ^####^ *p* < 0.0001 vs DW; ^A^ *p* < 0.05, ^C^ *p* < 0.001, and ^Z^ *p* > 0.05 vs N; ^δ^ *p* < 0.0001 and ^θ^ *p* > 0.05 vs Neg.

**Figure:**
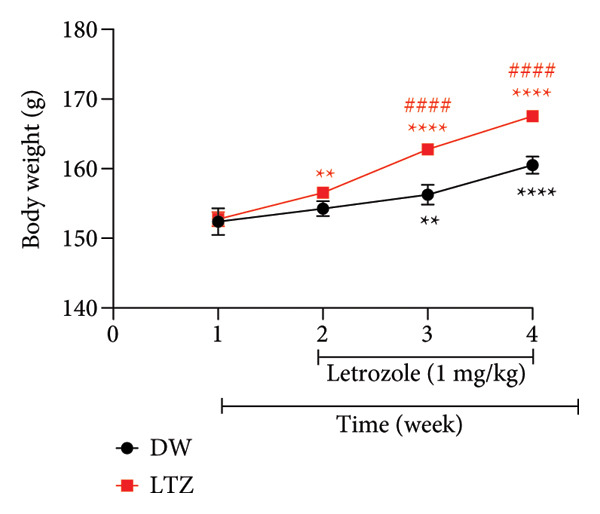
(a)

**Figure:**
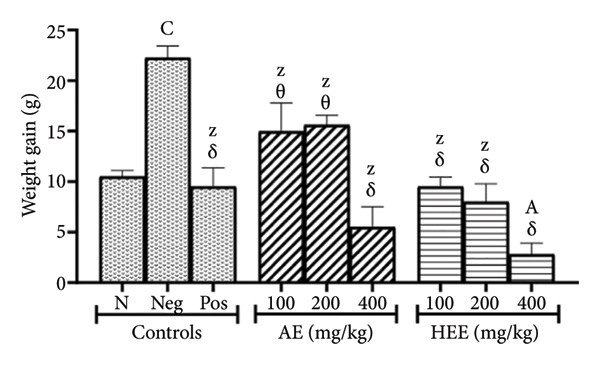
(b)

In the LTZ group, weight gain was greater over the weeks. It increased from 152.74 ± 1.03 g in the first week to 156.54 ± 0.41 g in the second week (2.48% increase; *p* = 0.0036), 162.78 ± 0.59 g in the third week (6.57% increase; *p* < 0.0001), and 167.55 ± 0.82 g in the fourth week (9.69% increase; *p* < 0.0001) (Figure 2(a)).

Compared to the DW group, body weight increased by 4.18% (from 156.25 ± 1.42 g in the DW group to 162.78 ± 0.59 g in the LTZ group; *p* < 0.0001) and by 4.39% (from 160.50 ± 1.22 g in the DW group to 167.55 ± 0.82 g in the LTZ group; *p* < 0.0001) after 2 and 3 weeks of LTZ administration, respectively (Figure 2(a)).

After treatment, body weight continued to increase in all animal groups except the negative control group, where the increase was significantly greater. A weight gain of 22.26 ± 1.17 g was observed in the negative control group, compared to 10.50 ± 0.59 g in the normal control group, representing a 112% increase (*p* = 0.0002) in the negative control group. Body weight gain in the positive control group decreased to 9.50 ± 1.87 g (a 57.32% decrease compared to the negative control group; *p* < 0.0001). In animals treated with the AE of *C. citratus*, body weight gain decreased at all doses tested: This parameter decreased from 22.26 ± 1.17 g in the negative control group to 14.97 ± 2.80 g at the dose of 100 mg/kg (i.e., a decrease of 32.75%; *p* = 0.0567), 15.60 ± 0.95 g at the dose of 200 mg/kg (i.e., a decrease of 29.92%; *p* = 0.1084), and 5.50 ± 2.01 g at the dose of 400 mg/kg (i.e., a decrease of 75.29%; *p* < 0.0001). Similarly, the HEE of *C. citratus* decreased weight gain at all doses tested, compared to the negative control: This parameter decreased to 9.50 ± 0.95 g at the dose of 100 mg/kg (i.e., a decrease of 57.32%; *p* < 0.0001), to 8.00 ± 1.77 g at the dose of 200 mg/kg (i.e., a decrease of 64.06%; *p* < 0.0001), and to 2.80 ± 1.10 g at the dose of 400 mg/kg (i.e., a decrease of 87.42%; *p* < 0.0001) (Figure 2(b)).

### 3.4. Effects on Serum Testosterone and Estradiol Levels

Serum testosterone levels were 1.24 ± 0.06 ng/mL in the normal control group versus 1.81 ± 0.05 ng/mL in the negative control group, representing an increase of 45.97% (*p* < 0.0001). Metformin decreased serum testosterone levels to 1.39 ± 0.04 ng/mL, a decrease of 23.20% (*p* = 0.0005) compared to the negative control group. The AE of *C. citratus* induced a similar effect by reducing serum testosterone levels to 1.34 ± 0.07 ng/mL at the dose of 100 mg/kg (a decrease of 25.85% compared to the negative control; *p* < 0.0001), to 1.56 ± 0.09 ng/mL at the dose of 200 mg/kg (a decrease of 13.51% compared to the negative control; *p* = 0.1310), and to 1.22 ± 0.04 ng/mL at the dose of 400 mg/kg (a decrease of 32.65% compared to the negative control; *p* < 0.0001). Similarly, the HEE of *C. citratus* decreased this parameter to 1.13 ± 0.004 ng/mL at the dose of 100 mg/kg (a decrease of 37.10% compared to the negative control; *p* < 0.0001), to 1.30 ± 0.06 ng/mL at the dose of 200 mg/kg (a decrease of 28.02% compared to the negative control; *p* < 0.0001), and to 1.33 ± 0.07 ng/mL at the dose of 400 mg/kg (a decrease of 26.74% compared to the negative control; *p* < 0.0001) (Figure 3(a)).

Figure 3Effects of *C. citratus* extracts on serum testosterone (a) and estradiol (b) levels. AE: aqueous extract; HEE: hydroethanolic extract; N: normal control; Neg: negative control; Pos: positive control. The results are presented as mean ± SEM, *n* = 6; ^A^ *p* < 0.05, ^D^ *p* < 0.0001, and ^Z^ *p* > 0.05 vs N; ^α^ *p* < 0.05, ^β^ *p* < 0.01, ^χ^ *p* < 0.001, ^δ^ *p* < 0.0001, and ^θ^ *p* > 0.05 vs Neg.

**Figure:**
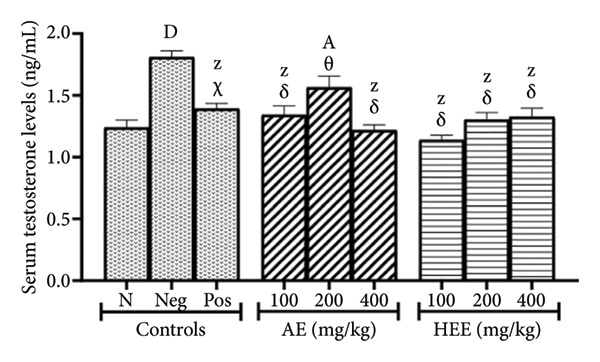
(a)

**Figure:**
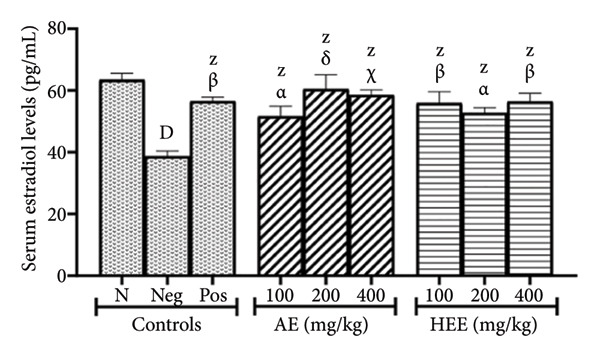
(b)

Figure 3(b) shows that serum estradiol levels decreased by 38.95% (*p* < 0.0001) in the negative control group (where serum estradiol levels were 38.73 ± 1.71 pg/mL) compared to the normal control group (where serum estradiol levels were 63.44 ± 2.07 pg/mL). After metformin treatment, serum estradiol levels increased by 45.95% (*p* = 0.0011) compared to the negative control group, reaching 56.53 ± 1.32 pg/mL. The AE of *C. citratus* induced a similar effect by increasing serum estradiol levels to 51.61 ± 3.28 pg/mL at the dose of 100 mg/kg (an increase of 33.26% compared to the negative control; *p* = 0.0415), to 60.48 ± 4.56 pg/mL at the dose of 200 mg/kg (an increase of 56.15% compared to the negative control; *p* < 0.0001), and to 58.53 ± 1.67 pg/mL at the dose of 400 mg/kg (an increase of 51.12% compared to the negative control; *p* = 0.0002). Similarly, the HEE of *C. citratus* increased serum estradiol levels to 55.90 ± 3.70 pg/mL at the dose of 100 mg/kg (an increase of 44.33% compared to the negative control; *p* = 0.0017), to 52.70 ± 1.69 pg/mL at the dose of 200 mg/kg (an increase of 36.07% compared to the negative control; *p* = 0.0198), and to 56.38 ± 2.73 pg/mL at the dose of 400 mg/kg (an increase of 45.57% compared to the negative control; *p* = 0.0012).

### 3.5. Effects on Serum Levels of GHRL and LEP

Figure 4(a) shows that serum GHRL levels were 33.36 ± 2.34 ng/mL in the normal control group versus 13.91 ± 1.02 ng/mL in the negative control group, representing a decrease of 58.30% (*p* < 0.0001). Metformin increased serum GHRL levels to 26.06 ± 1.49 ng/mL, an increase of 87.35% (*p* = 0.0006), compared to the negative control group. The AE of *C. citratus* induced a similar effect by increasing serum GHRL levels to 22.96 ± 2.14 ng/mL at the dose of 100 mg/kg (an increase of 65.06% compared to the negative control group; *p* = 0.0233), to 20.65 ± 1.80 ng/mL at the dose of 200 mg/kg (an increase of 48.45% compared to the negative control group; *p* = 0.1971) and to 31.91 ± 0.64 ng/mL at the dose of 400 mg/kg (an increase of 129.40% compared to the negative control group; *p* < 0.0001). Similarly, the HEE of *C. citratus* increased serum GHRL levels to 24.79 ± 2.09 ng/mL at the dose of 100 mg/kg (an increase of 78.22% compared to the negative control group; *p* = 0.0030), to 18.24 ± 0.99 ng/mL at the dose of 200 mg/kg (an increase of 31.13% compared to the negative control group; *p* = 0.7439), and to 27.51 ± 2.62 ng/mL at the dose of 400 mg/kg (an increase of 97.77% compared to the negative control group; *p* < 0.0001).

Figure 4Effects of *C. citratus* extracts on serum ghrelin (a) and leptin (b) levels. AE: aqueous extract; HEE: hydroethanolic extract; N: normal control; Neg: negative control; Pos: positive control. Results are presented as mean ± SEM. *n* = 6; ^A^ *p* < 0.05, ^B^ *p* < 0.01, ^C^ *p* < 0.001, ^D^ *p* < 0.0001, and ^Z^ *p* > 0.05 vs N; ^α^ *p* < 0.05, ^β^ *p* < 0.01, ^χ^ *p* < 0.001, ^δ^ *p* < 0.0001, and ^θ^ *p* > 0.05 vs Neg.

**Figure:**
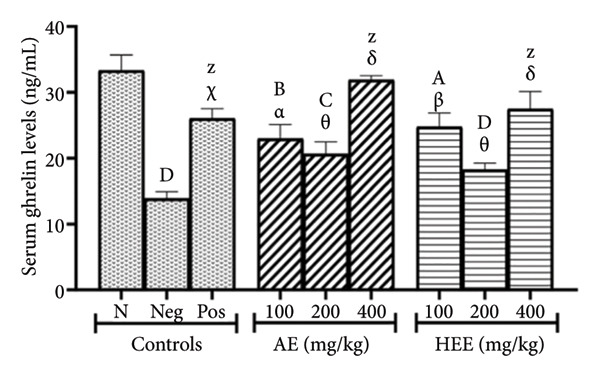
(a)

**Figure:**
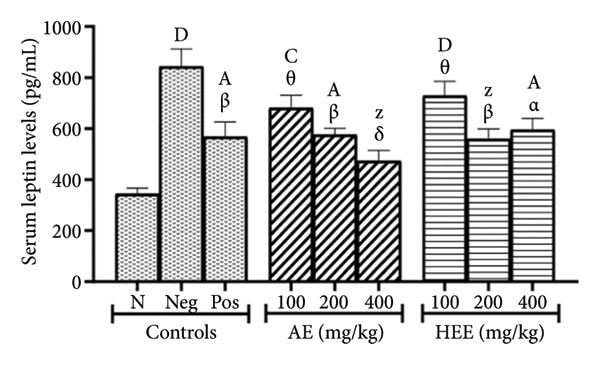
(b)

Serum LEP levels were 343.83 ± 22.75 pg/mL in the normal control group versus 843.35 ± 67.83 pg/mL in the negative control group, representing an increase of 145.28% (*p* < 0.0001). Metformin decreased serum LEP levels to 567.69 ± 58.58 pg/mL, a decrease of 32.69% (*p* = 0.0044) compared to the negative control group. AE of *C. citratus* induced a similar effect by decreasing serum LEP levels in a dose‐dependent manner. The extract in question induced serum LEP levels of 681.52 ± 49.49 pg/mL at the dose of 100 mg/kg (a decrease of 19.19% compared to the negative control; *p* = 0.2936), of 575.79 ± 25.33 pg/mL at the dose of 200 mg/kg (a decrease of 31.73% compared to the negative control; *p* = 0.0063), and of 473.47 ± 41.05 pg/mL at the dose of 400 mg/kg (a decrease of 43.86% compared to the negative control; *p* < 0.0001). Similarly, the HEE of *C. citratus* decreased serum LEP levels to 729.36 ± 55.19 pg/mL at the dose of 100 mg/kg (a decrease of 13.52% compared to the negative control; *p* = 0.7365), to 559.48 ± 39.53 pg/mL at the dose of 200 mg/kg (a decrease of 33.66% compared to the negative control; *p* = 0.0030), and to 595.17 ± 45.03 pg/mL at the dose of 400 mg/kg (a decrease of 29.43% compared to the negative control; *p* = 0.0144) (Figure 4(b)).

### 3.6. Effects on Markers of Ferroptosis and Apoptosis in Ovaries of PCOS Animals

The ferroptosis level in granulosa cells was estimated by measuring ovarian Fe^2+^. This parameter was 0.06 ± 0.01 mg/g of protein in the normal control versus 0.10 ± 0.01 mg/g of protein in the negative control, representing a 66.67% increase (*p* = 0.0007) in the negative control. Metformin decreased ovarian Fe^2+^ levels to 0.08 ± 0.00 mg/g of protein, a 20% decrease (*p* = 0.0780) compared to the negative control group. The AE of *C. citratus* induced a similar effect by decreasing ovarian Fe^2+^ levels at the tested doses. This parameter therefore decreased to 0.08 ± 0.01 mg/g of protein at the dose of 100 mg/kg (a decrease of 20% compared to the negative control; *p* = 0.1325), to 0.07 ± 0.00 mg/g of protein at the dose of 200 mg/kg (a decrease of 30% compared to the negative control; *p* = 0.0273), and to 0.07 ± 0.00 mg/g of protein at the dose of 400 mg/kg (a decrease of 30% compared to the negative control; *p* = 0.0106). Similarly, the HEE of *C. citratus* decreased ovarian Fe^2+^ concentrations to 0.08 ± 0.00 mg/g of protein at the dose of 100 mg/kg (a 20% decrease compared to the negative control; *p* = 0.1208), to 0.07 ± 0.00 mg/g of protein at the dose of 200 mg/kg (a 30% decrease compared to the negative control; *p* = 0.0011), and to 0.07 ± 0.00 mg/g of protein at the dose of 400 mg/kg (a 30% decrease compared to the negative control; *p* = 0.0104) (Table 4).

**Table 4:** Effects of *C. citratus* extracts on ovarian levels of Fe^2+^ and caspase 3

Groups	Fe^2+^ (mg/g prot.)	Caspase 3 (ng/g prot.)
N	0.06 ± 0.01	9.80 ± 0.71
Neg	0.10 ± 0.01^c^	13.48 ± 0.71^b^
Pos	0.08 ± 0.00^Ζ,θ^	10.40 ± 0.77^Ζ,α^

AE100	0.08 ± 0.01^Ζ,θ^	11.43 ± 0.75^Ζ,θ^
AE200	0.07 ± 0.00^Ζ,α^	11.20 ± 0.93^Ζ,θ^
AE400	0.07 ± 0.00^Ζ,α^	11.99 ± 0.08^Ζ,θ^

HEE100	0.08 ± 0.00^Ζ,θ^	12.21 ± 0.38^Ζ,θ^
HEE200	0.07 ± 0.00^Ζ,β^	11.81 ± 0.32^Ζ,θ^
HEE400	0.07 ± 0.00^Ζ,α^	10.18 ± 0.55^Ζ,α^

*Note:* Fe^2+^, ferrous iron; HEE, hydroethanolic extract; N, normal control; Neg, negative control; Pos, positive control; prot., protein. Results are presented as mean ± SEM., *n* = 6.

Abbreviation: AE, aqueous extract.

^b^ *p* < 0.01.

^c^ *p* < 0.001.

^Z^ *p* > 0.05 vs N.

^α^ *p* < 0.05.

^β^ *p* < 0.01.

^θ^ *p* > 0.05 vs Neg.

Granulosa cell apoptosis was assessed by measuring ovarian caspase 3 levels. The ovarian level of this parameter was 9.80 ± 0.71 ng/g of protein in the normal control group versus 13.48 ± 0.71 ng/g of protein in the negative control group, representing a 37.55% increase (*p* = 0.0046) in the latter. The metformin‐induced ovarian caspase 3 level was 10.40 ± 0.77 ng/g of protein, a 22.85% decrease (*p* = 0.0304) compared to the negative control group. AE of *C. citratus* induced a similar effect at the tested doses, although this effect was not statistically significant. This extract decreased ovarian caspase 3 levels to 11.43 ± 0.75 ng/g of protein at the dose of 100 mg/kg (a decrease of 15.21% compared to the negative control; *p* = 0.3635), to 11.20 ± 0.93 ng/g of protein at the dose of 200 mg/kg (a decrease of 16.91% compared to the negative control; *p* = 0.2361), and to 11.99 ± 0.08 ng/g of protein at the dose of 400 mg/kg (a decrease of 11.05% compared to the negative control; *p* = 0.7583). A similar effect was induced by the HEE of *C. citratus* at the tested doses. The said extract decreased ovarian caspase 3 levels to 12.21 ± 0.38 ng/g of protein at the dose of 100 mg/kg (a decrease of 9.42% compared to the negative control; *p* = 0.8816), to 11.81 ± 0.32 ng/g of protein at the dose of 200 mg/kg (a decrease of 12.39% compared to the negative control; *p* = 0.6350), and to 10.18 ± 0.55 ng/g of protein at the dose of 400 mg/kg (a decrease of 24.48% compared to the negative control; *p* = 0.0156) (Table 4).

### 3.7. Effects on Markers of Oxidative Stress in Ovaries of PCOS Animals

Oxidative stress was assessed by measuring ovarian levels of MDA, catalase, reduced GSH, and sulfur dioxide (SO_2_).

Ovarian MDA levels increased from 19.44 ± 0.90 mmol/mg protein in the normal control group to 26.71 ± 0.64 mmol/mg protein in the negative control group, representing a 37.40% increase (*p* = 0.0033). Metformin decreased ovarian MDA levels to 19.48 ± 2.37 mmol/mg protein, a 27.07% decrease (*p* = 0.0035) compared to the negative control group. Only the AE of *C. citratus* induced an effect similar to that of metformin at the dose of 100 mg/kg, with a 23.29% decrease (*p* = 0.0192) in ovarian MDA (20.49 ± 1.05 mmol/mg protein) compared to the negative control group. The two other doses of this extract decreased this parameter by 4.46% at the dose of 200 mg/kg (the induced MDA level was 25.52 ± 0.93 mmol/mg protein; *p* = 0.9986) and by 11.16% at the dose of 400 mg/kg (the induced MDA level was 23.73 ± 0.88 mmol/mg protein; *p* = 0.7227), compared to the negative control. The HEE of *C. citratus* also induced a slight decrease in ovarian MDA levels at the tested doses: a decrease of 10.07% at the dose of 100 mg/kg (the induced MDA level was 24.02 ± 1.01 mmol/mg of protein; *p* = 0.8187), a decrease of 8.39% at the dose of 200 mg/kg (the induced MDA level was 24.47 ± 0.87 mmol/mg of protein; *p* = 0.9245), and a decrease of 9.77% at the dose of 400 mg/kg (the induced MDA level was 24.10 ± 1.38 mmol/mg of protein; *p* = 0.8403), compared to the negative control (Table 5).

**Table 5:** Effects of *C. citratus* extracts on ovarian levels of some oxidative stress parameters.

Groups	MDA (mmol/mg prot.)	Catalase (U/mg prot.)	GSH (μmol/mg prot.)	SO_2_ (mmol/mg prot.)
NC	19.44 ± 0.90	73.48 ± 3.49	47.18 ± 2.17	5.57 ± 0.22
NegC	26.71 ± 0.64^b^	68.58 ± 2.88^Ζ^	32.76 ± 0.93^b^	8.98 ± 0.30^d^
PosC	19.48 ± 2.37^Ζ,β^	80.86 ± 4.52^Ζ,θ^	45.34 ± 3.65^Ζ,β^	5.73 ± 0.11^Ζ,δ^

AE100	20.49 ± 1.05^Ζ,α^	84.23 ± 4.46^Ζ,θ^	46.18 ± 1.03^Ζ,β^	7.22 ± 0.58^Ζ,θ^
AE200	25.52 ± 0.93^a,θ^	75.21 ± 1.15^Ζ,θ^	42.44 ± 2.78^Ζ,θ^	6.24 ± 0.26^Ζ,β^
AE400	23.73 ± 0.88^Ζ,θ^	86.74 ± 6.19^Ζ,θ^	44.27 ± 1.36^Ζ,α^	6.72 ± 0.60^Ζ,α^

HEE100	24.02 ± 1.01^Ζ,θ^	89.14 ± 5.58^Ζ,α^	40.48 ± 1.60^Ζ,θ^	6.70 ± 0.46^Ζ,α^
HEE200	24.47 ± 0.87^Ζ,θ^	81.97 ± 4.28^Ζ,θ^	46.65 ± 3.50^Ζ,β^	6.28 ± 0.36^Ζ,β^
HEE400	24.10 ± 1.38^Ζ,θ^	83.25 ± 3.75^Ζ,θ^	39.84 ± 1.03^Ζ,θ^	7.82 ± 0.58^a,θ^

*Note:* GSH, glutathione; HEE, hydroethanolic extract; MDA, malondialdehyde; N, normal control; Neg, negative control; Pos, positive control; prot., protein; SO_2_, sulfur dioxide. Results are presented as mean ± SEM., *n* = 6.

Abbreviation: AE, aqueous extract.

^a^ *p* < 0.05.

^b^ *p* < 0.01.

^d^ *p* < 0.0001.

^Z^ *p* > 0.05 vs N.

^α^ *p* < 0.05.

^β^ *p* < 0.01.

^δ^ *p* < 0.0001.

^θ^ *p* > 0.05 vs Neg.

Catalase levels decreased from 73.48 ± 3.49 U/mg of protein in the normal control group to 68.58 ± 2.88 U/mg of protein in the negative control group, a decrease of 6.67% (*p* = 0.9959). Metformin increased this parameter to 80.86 ± 4.52 U/mg of protein, an increase of 17.91% (*p* = 0.5287) compared to the negative control group. The AE of *C. citratus* induced a similar effect at doses of 100 mg/kg (induced catalase level was 84.23 ± 4.46 U/mg protein; an increase of 22.82%; *p* = 0.2187) and 400 mg/kg (induced catalase level was 86.74 ± 6.19 U/mg protein; an increase of 26.48%; *p* = 0.0911), compared to the negative control group. A similar effect was observed with the HEE of *C. citratus* at the doses tested: an increase of 29.98% at the dose of 100 mg/kg (induced catalase level of 89.14 ± 5.58 U/mg of protein; *p* = 0.0342), an increase of 19.52% at 200 mg/kg (induced catalase level of 81.97 ± 4.28 U/mg of protein; *p* = 0.4123), and an increase of 21.39% at 400 mg/kg (induced catalase level of 83.25 ± 3.75 U/mg of protein; *p* = 0.2933), compared to the negative control group (Table 5).

Ovarian reduced GSH levels decreased from 47.18 ± 2.17 μmol/mg of protein in the normal control group to 32.76 ± 0.93 μmol/mg of protein in the negative control group, a decrease of 30.56% (*p* = 0.0013). Metformin increased ovarian GSH levels to 45.34 ± 3.65 μmol/mg of protein (an increase of 38.40% compared to the negative control group; *p* = 0.0074). The AE of *C. citratus* induced a similar effect by increasing ovarian GSH levels to 46.18 ± 1.03 μmol/mg of protein at the dose of 100 mg/kg (an increase of 40.96% compared to the negative control; *p* = 0.0034), 42.44 ± 2.78 μmol/mg of protein at the dose of 200 mg/kg (an increase of 29.55% compared to the negative control; *p* = 0.0834), and 44.27 ± 1.36 μmol/mg of protein at the dose of 400 mg/kg (an increase of 35.13% compared to the negative control; *p* = 0.0193). The HEE also increased ovarian GSH levels at the doses tested: an increase of 23.57% at the dose of 100 mg/kg (induced GSH levels: 40.48 ± 1.60 μmol/mg protein; *p* = 0.2938), of 42.40% at the dose of 200 mg/kg (induced GSH levels: 46.65 ± 3.50 μmol/mg protein; *p* = 0.0022), and of 21.61% at the dose of 400 mg/kg (induced GSH levels: 39.84 ± 1.03 μmol/mg protein; *p* = 0.4058), compared to the negative control group (Table 5).

Ovarian SO_2_ levels were 5.57 ± 0.22 mmol/mg of protein in the normal control group versus 8.98 ± 0.30 mmol/mg of protein in the negative control group, representing a 61.22% increase (*p* < 0.0001) in the latter group. Metformin lowered this parameter to 5.73 ± 0.11 mmol/mg of protein, a 36.19% decrease (*p* < 0.0001) compared to the negative control group. The AE of *C. citratus* induced a similar effect by decreasing ovarian SO_2_ levels at the tested doses, compared to the negative control group. After treatment with this extract, ovarian SO_2_ decreased to 7.22 ± 0.58 mmol/mg of protein at the dose of 100 mg/kg (a decrease of 19.60% compared to the negative control; *p* = 0.1009), to 6.24 ± 0.26 mmol/mg of protein at the dose of 200 mg/kg (a decrease of 30.51% compared to the negative control; *p* = 0.0010) and to 6.72 ± 0.60 mmol/mg of protein at the dose of 400 mg/kg (a decrease of 25.17% compared to the negative control; *p* = 0.0121). Similarly, the HEE of *C. citratus* decreased ovarian SO_2_ levels to 6.70 ± 0.46 mmol/mg of protein at the dose of 100 mg/kg (a decrease of 25.39% compared to the negative control; *p* = 0.0110), to 6.28 ± 0.36 mmol/mg of protein at the dose of 200 mg/kg (a decrease of 30.07% compared to the negative control; *p* = 0.0013), and to 7.82 ± 0.58 mmol/mg of protein at the dose of 400 mg/kg (a decrease of 12.92% compared to the negative control; *p* = 0.5865) (Table 5).

### 3.8. Effects on the Histomorphology of the Ovaries

**Figure 6:**
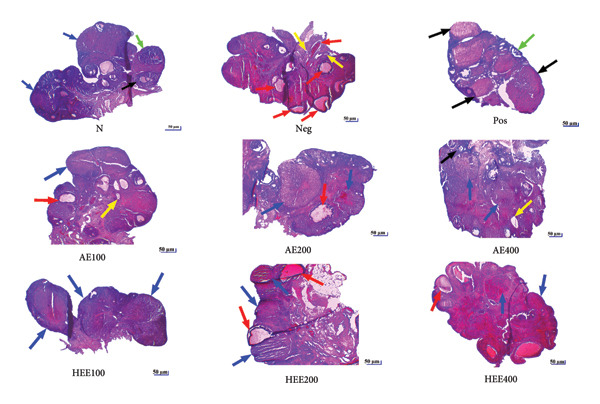
Microphotographs (X 40, hematoxylin and eosin) of ovaries of experimental animals. AE: aqueous extract; HEE: hydroethanolic extract; N: normal control; Neg: negative control; Pos: positive control; Black arrow: tertiary follicle (follicle with a fluid‐filled cavity called the antrum, a central oocyte, and several layers of follicular cells); green arrow: Graafian follicle (the largest follicle with a well‐developed antrum and a peripheral oocyte surrounded by the *cumulus oophorus*); red arrow: cystic follicle (fluid‐filled cavity without oocyte); yellow arrow: atretic follicle (malformed follicle exhibiting degenerative cells); blue arrow: *Corpora lutea* (cluster of cells located at the periphery of the ovary).

The histological analysis of the ovaries essentially consisted of the identification and enumeration of the types of follicles present on the ovarian sections. These follicles were mainly tertiary follicles, Graafian follicles, *corpora lutea*, cystic follicles, and atretic follicles (Figures 5 and 6).

Figure 5Effects of *C. citratus* extracts on growth and maturation of ovarian follicles: Tertiary follicles (a), Graafian follicles (b), *corpora lutea* (c), cystic follicles (d), and atretic follicles (e). AE: aqueous extract; HEE: hydroethanolic extract; N: normal control; Neg: negative control; Pos: positive control; Results are presented as mean ± SEM. *n* = 6; ^A^ *p* < 0.05, ^B^ *p* < 0.01, ^C^ *p* < 0.001, ^D^ *p* < 0.0001, and ^Z^ *p* > 0.05 vs N; ^α^ *p* < 0.05, ^β^ *p* < 0.01, ^χ^ *p* < 0.001, ^δ^ *p* < 0.0001, and ^θ^ *p* > 0.05 vs Neg.

**Figure:**
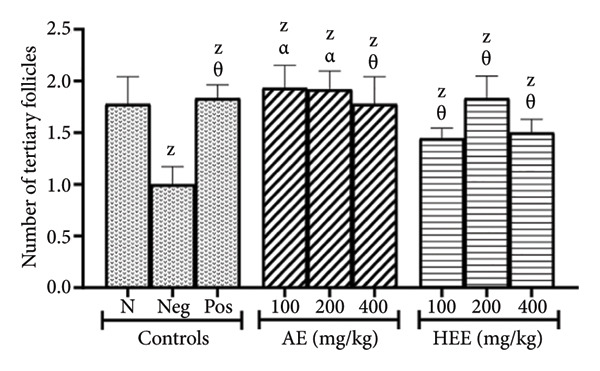
(a)

**Figure:**
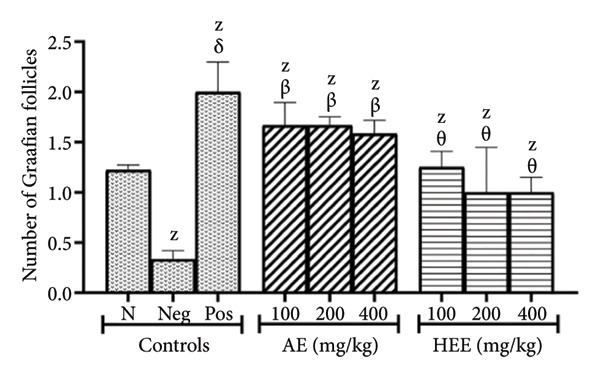
(b)

**Figure:**
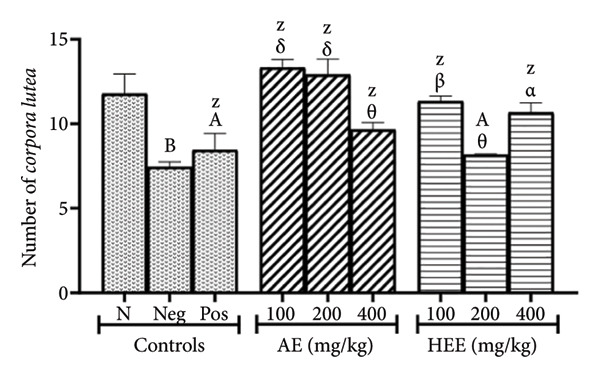
(c)

**Figure:**
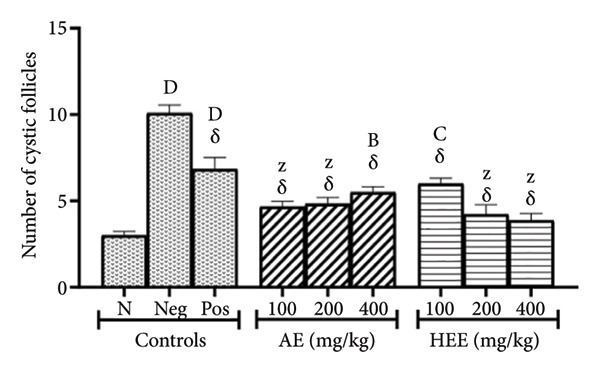
(d)

**Figure:**
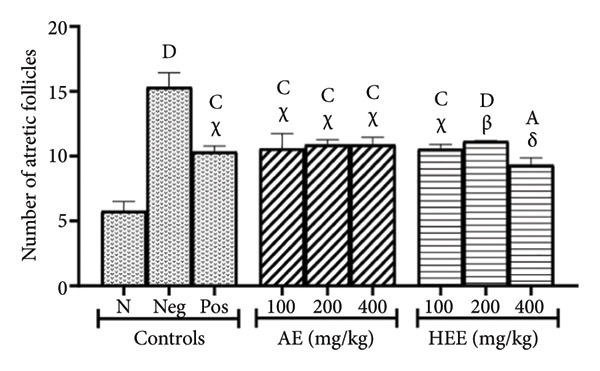
(e)

The results show that in the negative control group, the number of tertiary follicles was 1.00 ± 0.17, compared to 1.78 ± 0.26 in the normal control group, representing a 43.82% reduction (*p* = 0.1318) in the negative control group. Metformin increased the number of tertiary follicles by 83% (the number of induced follicles was 1.83 ± 0.13; *p* = 0.0836), compared to the negative control group. The AE of *C. citratus* induced a similar effect by increasing the number of tertiary follicles by 93% at the dose of 100 mg/kg (the number of follicles induced was 1.93 ± 0.22; *p* = 0.0339), by 92% at the dose of 200 mg/kg (the number of follicles induced was 1.92 ± 0.18; *p* = 0.0397), and by 78% at the dose of 400 mg/kg (the number of follicles induced was 1.78 ± 0.26; *p* = 0.1318), compared to the negative control. Similarly, the HEE of *C. citratus* increased the number of tertiary follicles by 44% at the dose of 100 mg/kg (the number of follicles induced was 1.44 ± 0.09; *p* = 0.7870), by 83% at the dose of 200 mg/kg (the number of follicles induced was 1.83 ± 0.21; *p* = 0.0836), and by 50% at the dose of 400 mg/kg (the number of follicles induced was 1.50 ± 0.13; *p* = 0.6650), compared to the negative control (Figure 5(a)).

The number of Graafian follicles was 0.33 ± 0.09 in the negative control group, compared to 1.22 ± 0.05 in the normal control group, representing a reduction of 72.95% (*p* = 0.1160) in the negative control group. Metformin induced a sixfold increase in the number of Graafian follicles (the number of follicles induced was 2.00 ± 0.30; *p* < 0.0001), compared to the negative control group. The AE of *C. citratus* induced a similar effect by causing a 5‐fold increase in the number of Graafian follicles at doses of 100 mg/kg (the number of follicles induced was 1.67 ± 0.23; *p* = 0.0023) and 200 mg/kg (the number of follicles induced was 1.67 ± 0.09; *p* = 0.0023), and a 4.79‐fold increase in the number of Graafian follicles at the dose of 400 mg/kg (the number of follicles induced was 1.58 ± 0.13; *p* = 0.0052), compared to the negative control group. Similarly, the HEE of *C. citratus* induced a 3.79‐fold increase in the number of Graafian follicles at the dose of 100 mg/kg (the number of follicles induced was 1.25 ± 0.16; *p* = 0.0947) and a 3‐fold increase in the number of Graafian follicles at doses of 200 mg/kg (the number of follicles induced was 1.00 ± 0.45; *p* = 0.4387) and 400 mg/kg (the number of follicles induced was 1.00 ± 0.15; *p* = 0.4387), compared to the negative control group (Figure 5(b)).

The number of *corpora lutea* counted in the ovaries of the negative control group was 7.44 ± 0.30, compared to 11.78 ± 1.69 in the normal control group, representing a 36.84% reduction (*p* = 0.0012) in the negative control group. After metformin treatment, the number of *corpora lutea* increased to 8.44 ± 0.98, a 13.44% increase (*p* = 0.9784) compared to the negative control group. The AE of *C. citratus* significantly increased the number of *corpora lutea* in the ovaries of treated rats. The rates of increase in these follicles induced by this extract were 79.17% at the dose of 100 mg/kg (the number of follicles induced was 13.33 ± 0.48; *p* < 0.0001), 73.66% at the dose of 200 mg/kg (the number of follicles induced was 12.92 ± 0.92; *p* < 0.0001), and 29.97% at the dose of 400 mg/kg (the number of follicles induced was 9.67 ± 0.41; *p* = 0.3456), compared to the negative control group. Similarly, the HEE of *C. citratus* increased the number of *corpora lutea* by 52.28% at the dose of 100 mg/kg (the number of induced follicles was 11.33 ± 0.31; *p* = 0.0052), by 9.81% at the dose of 200 mg/kg (the number of induced follicles was 8.17 ± 0.04; *p* = 0.9975), and by 43.41% at the dose of 400 mg/kg (the number of induced follicles was 10.67 ± 0.56; *p* = 0.0365), compared to the negative control group (Figure 5(c)).

In the ovaries of the negative control group, the number of cystic follicles was 10.08 ± 0.47, compared to 3.00 ± 0.23 in the normal control group, representing a 3.36‐fold increase (*p* < 0.0001) in the negative control group. Metformin decreased the number of cystic follicles by 32.24% (the number of follicles induced was 6.83 ± 0.67; *p* < 0.0001), compared to the negative control group. The AE of *C. citratus* induced a similar effect by decreasing the number of cystic follicles by 53.67% at the dose of 100 mg/kg (the number of follicles induced was 4.67 ± 0.31; *p* < 0.0001), by 52.08% at the dose of 200 mg/kg (the number of follicles induced was 4.83 ± 0.36; *p* < 0.0001), and by 45.44% at the dose of 400 mg/kg (the number of follicles induced was 5.5 ± 0.32; *p* < 0.0001), compared to the negative control group. Similarly, the HEE of *C. citratus* decreased the number of cystic follicles by 40.48% at the dose of 100 mg/kg (the number of follicles induced was 6.00 ± 0.31; *p* < 0.0001), by 58.13% at the dose of 200 mg/kg (the number of follicles induced was 4.22 ± 0.55; *p* < 0.0001), and by 61.61% at the dose of 400 mg/kg (the number of follicles induced was 3.87 ± 0.41; *p* < 0.0001), compared to the negative control group (Figure 5(d)).

The number of atretic follicles was 15.33 ± 1.10 in the negative control group, compared to 5.78 ± 0.73 in the normal control group, representing a 2.65‐fold increase (*p* < 0.0001) in the negative control group. Metformin reduced the number of atretic follicles to 10.33 ± 0.45, a reduction of 32.62% (*p* = 0.0002) compared to the negative control group. The AE of *C. citratus* induced a similar effect by decreasing the number of atretic follicles by 30.98% at the dose of 100 mg/kg (the number of follicles induced was 10.58 ± 1.16; *p* = 0.0004), and by 28.96% at doses of 200 mg/kg (the number of follicles induced was 10.89 ± 0.39; *p* = 0.0010) and 400 mg/kg (the number of follicles induced was 10.89 ± 0.56; *p* = 0.0010), compared to the negative control group. Similarly, the HEE of *C. citratus* decreased the number of cystic follicles by 31.12% at the dose of 100 mg/kg (the number of follicles induced was 10.56 ± 0.35; *p* = 0.0003), by 27.14% at the dose of 200 mg/kg (the number of follicles induced was 11.17 ± 0.04; *p* = 0.0024), and by 39.14% at the dose of 400 mg/kg (the number of follicles induced was 9.33 ± 0.54; *p* < 0.0001), compared to the negative control group (Figure 5(e)).

## 4. Discussion

Ovarian dynamics (hormone production, folliculogenesis) is an altered process in the ovaries of women and animals with PCOS. Indeed, this endocrinopathy is characterized by hyperandrogenism, obesity, and ovarian dysfunction (folliculogenesis disorder, oligo/anovulation, follicular atresia, and cyst formation) [32, 82]. In animals with LTZ‐induced PCOS, increased serum levels of GHRL [32, 83] and LEP [32] were also observed.

In agreement with these reports, our results showed that LTZ induced overweight, an increase in androgens associated with a decrease in estrogen, and a decrease in serum GHRL levels associated with increased serum LEP levels. High number of atretic and cystic follicles was observed in the ovaries of animals exposed to LTZ. Indeed, chronic hyperandrogenism induces excessive release of GnRH from the hypothalamus and LH from the adenohypophysis [16]. In high amounts, androgens stimulate pancreatic cells to release insulin [84]. The resulting hyperinsulinemia increases the bioavailability of IGF‐1 [5]. IGF‐1 and insulin potentiate the effect of LH on ovarian follicle theca cells, thus promoting androgen production [7, 8]. Hyperinsulinemia and IGF‐1 also amplify the effect of LH on granulosa cells, stimulating by ricochet, early differentiation of these cells, follicular growth arrest, anovulation, cyst formation, and follicular atresia [9, 10, 48].

Treatment of PCOS animals with AE and HEE of *C. citratus* reduced body weight gain, decreased serum testosterone levels, and increased serum estradiol levels. To induce these effects, *C. citratus* would have abolished the inhibitory effect of LTZ on aromatase thus promoting the conversion of androgens (including testosterone) into estrogens (including estradiol). Decreased testosterone levels by *C. citratus* would have contributed to reduced body weight gain, given the involvement of hyperandrogenism in lipogenesis, adipose tissue accumulation, and visceral obesity [85]. Hyperandrogenism is also known to reduce GHRL production [32] and increase LEP production [32, 86]. Indeed, GHRL is known for its ability to inhibit the activity of the hypothalamic–pituitary–ovarian axis because it inhibits the secretion of GnRH by the hypothalamic neurons, LH by the adenohypophysis, and testosterone by the ovaries [30]. GHRL also inhibits insulin production in PCOS animals [87]. It is known that, in the case of hyperandrogenism, the increased release of GnRH stimulates a consequent production of LH by the adenohypophysis. The latter (LH) consequently stimulates ovarian androgen production [16, 32, 88]. Insulin, in conjunction with IGF‐1, accentuates the steroidogenic effect of LH on ovarian follicle theca cells, thus maintaining hyperandrogenism [7, 8]. The increase in GHRL levels would have therefore contributed to an indirect decrease in serum testosterone levels in animals treated with *C. citratus* extracts. GHRL also contributes to follicular maturation by preventing apoptosis [31, 32]. Regarding LEP, the literature reports that it stimulates, at physiological rates, follicular development and steroidogenesis, but in high quantities, it increases testosterone secretion and promotes the formation of ovarian cysts [24, 25]. By bringing serum LEP levels back to normal and increasing those of GHRL, *C. citratus* inhibited the stimulus (hyperandrogenism) that dictates cyst formation in favor of ovarian follicle development and maturation.

PCOS‐related ovarian dysfunction has also been associated, in part, with increased programmed death of granulosa cells [11, 89]. Granulosa cell apoptosis plays a major role in follicular atresia [14–17]. The latter (granulosa cell apoptosis) is also considered a mark of follicular atresia [90]. Additionally, authors reported that nonapoptotic programmed cell death such as autophagy can be activated in granulosa cells during follicular atresia [89]. Ferroptosis is an autophagic cell death process characterized by iron (Fe2+)‐dependent lipid peroxidation and increased cellular ROS [18, 19, 91]. ROS, including free radicals such as superoxide (O_2_), hydroxyl radical (HO‐), and hydrogen peroxide (H_2_O_2_), are produced at high levels when cells suffer from environmental stress, damaging cellular components and triggering cell apoptosis [89]. Accumulation of ROS is considered one of the main causes of granulosa cell apoptosis [92, 93]. ROS have the potential to simultaneously trigger autophagia and apoptosis of granulosa cells [89]. This was marked in the present study by an increase in ovarian levels of MDA (a byproduct of lipid peroxidation and a marker of oxidative stress) in the PCOS control (negative control), an increase in ovarian levels of Fe2+ (marker of ferroptosis), and caspase 3 (marker of apoptosis). Overproduction of ROS in follicles is known to block follicle development, leading to follicular atresia [89]. The increase in SO_2_ and GSH levels in the ovaries of the negative control would be a response of the body to ovarian damage induced by PCOS, including oxidative stress (characterized by high levels of MDA) and cell death [characterized by high levels of Fe2+ (marker of ferroptosis) and caspase 3 (marker of apoptosis)] because SO_2_ and GSH are known to be antioxidant and antiapoptotic [26, 94].

*C. citratus* extracts would have inhibited death of ovarian follicle granulosa cells not only by lowering serum testosterone levels, but also by increasing those of estradiol. Estradiol is considered an antioxidant and an intraovarian cell survival factor [95–97]. The increase of this hormone by *C. citratus* extracts would be responsible, at least in part, for the improvement of ovarian damage induced by PCOS and the reduction of ROS and iron deposition in the ovaries of treated animals. Indeed, in addition to the increase in serum levels of estradiol in animals treated with extracts of *C. citratus*, a significant decrease in markers of ferroptosis (decrease in ovarian Fe2+ levels) and apoptosis (decrease in ovarian caspase 3 levels) was observed. These effects were associated with a decrease in ovarian levels of MDA and an increase in the scavengers of ROS (catalase and GSH), known as antioxidants. These results indicate a decrease in autophagy and apoptosis in ovarian follicles. Furthermore, there would be a synergistic action between estradiol and GHRL, both augmented by *C. citratus*, because GHRL is also known to promote follicular maturation, preventing apoptosis [31, 32]. Given that the autophagia and apoptosis of granulosa cells are markers of follicular atresia [90] and responsible for polycystic ovaries [52, 89, 98], all these events induced by *C. citratus* would have therefore contributed to follicular growth and maturation (marked by an increase in the number of tertiary and Graafian follicles), ovulation (marked by an increase in the number of *corpora lutea*), and the decrease of cystic and atretic follicles. In addition, thanks to its antioxidant potential, recently highlighted by Namekong et al. [56], *C. citratus* would also act directly on the ovaries of PCOS rats to trap ROS and correct tissue damage caused by these free radicals.

All these results show that the extracts of *C. citratus* are endowed with hypoandrogenic and gonadoprotective properties, which are mediated by the activation of aromatase (promoting the synthesis of estradiol), the simultaneous inhibition of autophagy (ferroptosis) and apoptosis of granulosa cells, and free radical trapping. These effects were also mediated by increased GHRL production and decreased LEP production. These effects induced by *C. citratus* extracts could be attributed to their chemical compositions. Indeed, polyphenols (tannins and flavonoids) are known to trap free radicals [61, 64]. Alkaloids are a class of chemical compounds with insulin‐sensitizing and antiapoptotic properties [65–67]. Through these insulin‐sensitizing properties, the alkaloids contained in the extracts of *C. citratus* would have decreased circulating levels of insulin, which would have prevented the amplifying activity of insulin on the effects of LH in ovarian follicles, thus decreasing ovarian production of testosterone and protecting ovarian follicles from follicular atresia and cyst formation. The results also show that the two extracts tested have an almost identical efficiency. Thus, the extraction methods carried out in this work (AE and HEE) would not have altered the quality of active ingredients and, consequently, the effectiveness of the plant. Although the level of alkaloids was significantly higher in the HEE, this did not influence the effectiveness of the said extract in comparison with the AE. This could be explained by the fact that alkaloids are effective even in small quantities [99]. Moreover, in pharmacology, it is known that when the dose–response curve reaches the maximal effect, regardless of the additional dose, the maximal effect remains unchanged [100, 101]. Thus, the AE would therefore have the minimum quantity of alkaloids necessary to induce a maximal effect; the reason why the 5‐times higher quantity of alkaloids in the HEE would not have modified the effectiveness of the plant. However, although the effectiveness of the HEE is similar to that of the AE, the abundance of alkaloids (5 times more) in the HEE questions the safety of the said extract because it is known that alkaloids are often toxic or dangerous to the body in high doses [102].

In conclusion, AE and HEE of *C. citratus* corrected PCOS‐induced ovarian follicular atresia in rats by reducing hyperandrogenism and modulating LEP and GHRL production. Although the extraction methods (AE and HEE) used in this study did not alter the plant’s efficacy, the AE of *C. citratus* appears ideal due to its low alkaloid content. Therefore, regular consumption of this extract could protect the ovaries from premature follicular exhaustion caused by pathologies such as PCOS. However, further research is needed to fully understand the therapeutic potential of *C. citratus* on PCOS‐induced ovarian lesions, focusing on the mechanisms by which *C. citratus* attenuates the ferroptosis and apoptosis underlying PCOS.

## Disclosure

All persons involved in the research and/or preparation of the manuscript have been listed as authors/co‐authors. All authors have accepted responsibility for the entire content of this manuscript and approved its submission.

## Conflicts of Interest

The authors declare no conflicts of interest.

## Author Contributions

Vanis Slauvers Akago, Marie Alfrede Mvondo, Sefirin Djiogue, Christophe Mezui, Hélène Carole Edima‐Durand, Jules‐Roger Kuiate, and Dieudonné Njamen: conceptualization, methodology, resources, project administration, supervision, data curation, formal analysis, validation, and writing–review and editing.

Vanis Slauvers Akago, Nina‐Sonia Messongue Mbollo, Agatha Yelah Ntumnyuy, Perpetue Atsama Mbede, Josue Jobin Biba, Lylie Gisèle Atsafack Mboudem, and Stephen Lacmata Tamekou: investigation, visualization, and writing–original draft.

## Funding

No funding was received for this research.

## Data Availability

The data that support the findings of this study are available from the corresponding author upon reasonable request.
